# Public–Medicine Dissonance: Why in a World of Evidence-based Medicine?

**DOI:** 10.5041/RMMJ.10226

**Published:** 2015-10-26

**Authors:** Michael Gordon

**Affiliations:** Baycrest Health Sciences, Toronto, Ontario, Canada

**Keywords:** Anti-vaccination movement, conspiracy theories, evidence-based medicine, medical quackery, trust in physicians

## Abstract

The evolution of medicine is quite remarkable and astounding. Modern medicine is successfully treating or providing long-term control of conditions which in the not-so-distant past were lethal or resulted in permanent disability. The strong emphasis on evidence-based medicine in today’s medical profession has led to a more organized approach toward evaluating the safety and efficacy of new medical treatments. Despite attempts to meet the complex needs of an ever-aging population, an almost cynical or inherent distrust of physicians in general and their medical claims is being increasingly noted. For many physicians this has led to an uncomfortable sense of professional frustration as doubt is cast on themselves or the medical profession in general when the expectations and goals of patients or their families are not achieved. The causes of this apparent malady of contemporary medicine are myriad and may be explored from various perspectives, depending on the particular issue. To understand better the issues and challenges involved, today’s medical practitioner needs to be aware of the complex mix of organizational, professional, ethical, and at times anthropological perspectives contributing to this dissonance between medical professionals and the public. Improving our insight into the forces at work in this dissonance will help medical professionals improve medical services to the public and contribute to the preservation of medicine’s admirable historical legacy.

## INTRODUCTION

The long history of medicine and its legacy has been filled with marvelous accomplishments. One miracle of medicine is the continuous increase in longevity from birth, even in developing countries.^1^ Over the centuries, physicians have generally engendered respect, and even adulation, from the general public and colleagues in academia and governing bodies. The awards for medical accomplishments and discoveries are myriad. The media’s portrayal of medical stories and the long list of books authored by physicians is a testimony to the general interest and reverence the public has generally held for medicine and its practitioners. It is therefore disquieting that there appears to be public disillusionment with medical practice and the profession in recent years. The media are increasingly exposing or discrediting the medical field and institutions with an almost missionary zeal, further contributing to the public’s disillusionment with medicine. The causes for this situation are many and complex. A better understanding of these issues will equip medical practitioners to serve the public better in their ethical and clinical mission.

Medical practice has been rapidly changing for many decades: for the practicing physician, including those in the forefront of academic medicine, it is challenging to keep up with what is referred to as evidence-based medicine (EBM). By this we generally mean medical knowledge/facts based on the best of current medical research as reported in reputable medical journals.^2^ One could characterize the basic characteristics of EBM by the criteria laid out by the New York University School of Medicine Medical Library definition:^3^

Evidence-based medicine asks questions, finds and appraises the relevant data, and harnesses that information for everyday clinical practice. Evidence-based medicine follows four steps:

- Formulate a clear clinical question from a patient’s problem
- Search the literature for relevant clinical articles
- Evaluate (critically appraise) the evidence for its validity and usefulness
- Implement useful findings in clinical practice^3^

This might be a very compelling approach particularly for academic physicians. However, there may be unexpected negative repercussions, due to the contemporary focus on “evidence” as the arbiter of competent and acceptable medical care. The focus on clinical outcomes and the reported morbidities and deaths from medical interventions reflects only part of the picture of modern medicine. Some have suggested that to produce a more sensitive and receptive physician, a more robust focus on the humanities in medicine might be necessary to counterbalance the general emphasis on the medical sciences.^4^ The rationale for medical decisions does not always resonate with the expectations and belief systems of patients and/or their families, as the following prototypical case demonstrates.

## PROTOTYPICAL COMPOSITE CASE

Mrs JL, an 88-year-old widow, had been ill for over a decade. A previously heavy smoker, she suffered for years from severe chronic obstructive pulmonary disease (COPD): despite quitting smoking 8 years previously, she experienced dyspnea on minimal exertion. She had two myocardial infarctions, one at age 82 and the second at age 84. Despite stenting she continued to experience angina and intermittent heart failure resulting in frequent hospital admissions, the last being 2 months before she died. She was admitted with florid pulmonary edema likely precipitated by a pulmonary tract infection. During treatment, Mrs JL was found to have moderate renal insufficiency. During the preceding 3 years she also had increasing cognitive impairment with a diagnosis of mixed dementia with MRI evidence of multiple small lacunar cerebral infarcts.

Mrs JL experienced a number of transfers to different apparently suitable facilities. However, over time she deteriorated and required repeated acute hospital admissions. Her devoted son decreased his workload as a solo lawyer in order to help with her care which also required the assistance of a personal support worker (PSW). While in a retirement home after one of her acute admissions with the constant companionship of the personal support worker, Mrs JL apparently aspirated some cereal. Despite attempts by the PSW to dislodge the obstruction it took the emergency medical service (EMS) some minutes to successfully do so; by this time she had sustained anoxic brain damage.

She was admitted to a general hospital, intubated in the emergency room, and transferred to the intensive care unit (ICU). Mrs JL did not regain consciousness and suffered a wide array of medical complications including infections, renal insufficiency, progressive cognitive impairment and delirium, and multi-system dysfunction. Various specialists were consulted on her care, with each one explaining to the son why an extreme intervention such as dialysis could not be considered.

The son, who was religiously devout, claimed that his views were also held by his ailing mother. He had great difficulty during conversations held with the ICU director and each of the sub-specialists regarding options for further care of his mother. When the ICU director advised that Mrs JL was actually in the terminal stage of multiple illnesses and recommended her transfer to a palliative care unit, the son reacted almost violently, accusing the physicians of “abandoning” his mother because “she was old” and not respecting their religious beliefs regarding the sanctity of life.

Despite conversations with the unit’s social worker, a meeting with the facility’s ethicist, and a meeting with a religious leader of his faith, the son was adamant in his denial of the seriousness of his mother’s illness. In an effort to provide an objective third-party opinion, an external consultant from another facility saw the patient and concurred with the recommendations regarding palliative care; the son dismissed him as being “in cahoots” with the medical staff. Eventually Mrs JL died, unconscious while still on the ventilator.

One month later the hospital’s regulatory body received a complaint from the son outlining all the possible deficiencies in his mother’s care and quoting extensively from internet-based sites that discussed possible interventions for each of the organ failures she had experienced. In his complaint he accused the attending and consulting doctors of incompetence, negligence, age-bias, and religious discrimination against his mother. The son demanded that the hospital be punished. In addition to the regulatory body complaint he initiated a civil suit and a complaint to the Human Rights Commission asking for significant damages based on the charge of “wrongful death and religious bias.” After review and deliberation, the regulatory body ruled against the complaint; the civil suit never materialized, and the Human Rights Commission rejected his claim.

## EVIDENCE-BASED MEDICINE: IS THE PUBLIC’S RESPONSE AN ANTHROPOLOGICAL ISSUE?

It is important to contemplate where EBM sits in the public mind. Medical professionals often ignore the fact that many in the public have no background in or elementary understanding of scientific concepts, or of the challenge to interpret medical research correctly. A person’s underlying educational knowledge base, culture, and belief system often colors their understanding of information conveyed by health care professionals regarding their or a loved one’s medical condition. Combined, these constitute the anthropological basis for attitudes and beliefs about medicine, and impact the public’s core responses to medical care.

### Example of Belief-systems that Contradict Evidence-based Medicine: The Anti-vaccination Movement

An example of the disconnect between the public and medical recommendations based on convincing medical evidence is reflected in the anti-vaccination movement. Despite the evidence available and presented to the public, this movement seems to fly in the face of logical decision-making. The anti-vaccination movement is a glaring example of how misinformation has been combined with a concepttual belief in conspiracy theories about how health care information is developed and then promoted.

Although compelling evidence has discredited the medical article published some years ago which proposed a link between childhood measles vaccination and autism, many people, including high-profile personalities, continue to dispute the retraction of the pro-autism article.^5^ Supporters of this movement propose conspiracies to promote vaccination because of the great financial benefits that are the motivation of “Big Pharma.” The anti-vaccination movement has recently become a high-profile media feast due to the measles outbreak among visitors to Disneyland and the related spread of measles throughout parts of the United States.^6^ There have also been outbreaks of other infectious diseases, which had not been seen in Western countries in recent years due to the almost universal vaccination programs. One recent outbreak of whooping cough among siblings was a game-changer when the parent realized that her anti-vaccine position placed her children at risk—she is now a vocal advocate for vaccination.^7^

The *anti-vaxxers* have a world view that might be classified as expressing an anthropological underpinning; it has for many superseded the important EBM and clinical cornerstones of public health and has imperiled the life and well-being of many children.

### Why Medical Evidence is Not Always Compelling

Medical practitioners in Western countries have been nurtured on the concept of, and almost deified, EBM. However, the public has been slow to understand the underpinnings of EBM, and physicians have not always been adept at using their evidence-based knowledge to the best advantage when explaining the complexities of medicine to patients, to the public, or to the media. Easy access to popular media outlets, including the ever-present internet and social media, acts as a complicit nay-sayer and critic of what physicians believe is the “best evidence” available. This is compounded by the failure to acknowledge the ever-changing landscape of medical knowledge, where today’s evidence can completely turn around and become last year’s erroneous medical premise.

The cultural and anthropological implications of a belief system founded on the science of medicine and the EBM methodology are that public skepticism is difficult to address, as is the skepticism of eminent physicians who remind the profession of the fluidity of EBM. A society built on the ideals of the scientific method and enlightened understanding of the world does not eliminate undercurrents that often reflect a variety of belief systems including religious tenets that may starkly oppose the best that science has to offer, exemplified by EBM.^8^

## CLINICALLY BASED DISAPPOINTMENTS IN MEDICAL CARE: PATIENT AND FAMILY PERSPECTIVES

During the last two decades I have been privileged to be involved in several organizations that review medical cases where a complaint was filed against physicians and/or the institution providing the medical care, as a result of a “bad” or “unexpected” patient outcome. Processing such complaints varies from jurisdiction to jurisdiction, but usually consists of a predominately legal process (in law the *tort* process), and a regulatory process by which a jurisdictional body is given the authority and responsibility to self-regulate and investigate complaints against physicians. The latter process does not exclude the use of the legal/tort system but in many instances obviates that route if the complaining member of the public is satisfied that a careful and credible review of the case has occurred.

The observations that I have made from a collection of observed cases have helped me to understand the process, the dynamics, and the motivation behind the complaint, as well as the responses of those against whom the complaint has been made. These types of cases often involve a range of issues, including the complainant’s belief systems, their understanding of the “science” of medicine, their faith in the organizational structure of medicine, and their trust in those providing the care—the physicians and/or institutions.

### Types of Complaints

There seem to be categories of complaints that are similar in nature. While the facts pertaining to each case may differ, the principles involved in the complaint are similar, focusing primarily on the treating physicians and/or the institutional setting and organization of care.

#### “Inadequate or Inappropriate Communication” or “Miscommunication”

One of the greatest causes of complaints by patients and families against physicians is related to communication.^9^,^10^ The complaints range from the “tone” of the communication; whether the physician appeared to have characteristics that might be described as “impatient,” “demeaning,” “dismissive,” “rude,” “insulting,” or, at the very extreme, “abusive.” When this is the major component of the complaint, in the absence of a reliable unbiased witness or some confirmatory or impartial source, it may be impossible to determine if there was anything amiss in communication. Even when there might be some documentation of the interchange, it may still be difficult to determine the nature of what transpired. For example, what might be construed by a patient or family as “yelling” might be explained as cultural or a communication style of the physician; for example, the decibel level could be related to what might be explained as impassioned speech. Family therapists often hear complaints by one spouse about an insulting or abusive method of speaking, only to receive a rebuttal that the other person’s tone of voice was related to their personal “style,” or social or cultural background, or their passionate speech because of the topic being discussed. The tone of speech is may sometimes be related to the emotional state of the person speaking. However, if it is a doctor, the tone of voice may have little to do with the actual clinical judgement being made. Therefore the “he said, she said” scenario is often difficult to substantiate as the basis of the complaint.

When reviewing complaints by patients or substitute decision-makers (SDMs; also known as a health care proxy in the USA) regarding a physician’s apparently poor communication, the problem often has less to do with the “facts” or the “information” actually given than it does with what the person perceived or took away from any conversation. A common example is when a physician is asked to prognosticate or predict the course of an illness. The physician may mention a time line, for example, “anywhere from 2 to 6 months.” However, the patient or SDM understands this to mean 6 months as the operative time; hence, should death occur prematurely the physician is accused of “misrepresenting the prognosis” or failing to fulfill expected medical treatments. Similar misinterpretations may occur when a physician trained in the world of medical statistics says something to the effect of “mean survival time.” What may not be understood by the patient or SDM is that this time period does not indicate what the patient in question is likely to achieve, but what a group of patients has been noted to achieve from statistical analyses. This may not represent the individual patient. Within the statistical context, this information may be part of the distribution of outcomes—but it is of no help to the SDM who is focusing on the idea of “an average” time and assumes that their loved one is likely to be in that “average” range. Some are critical of communication styles that focus on “facts” and the “average” rather than finding ways to focus on the individual patient with that patient’s unique characteristics and the relationship with their family. Indeed, this is what many family members are looking for from their physicians, especially when the discussions are of a serious nature.^11^,^12^

The degree of reaction to such events can be very challenging for physicians who in their own mind believe they have been quite accurate in their prognostic efforts, only to be accused of “lying” or of inadequately communicating the patient’s true condition. In some cases, the reality is that no matter how carefully the information was provided, the negative or apparently premature outcome becomes the focus of the family’s concern and/or complaint.

#### Failure to Pursue All Potential Avenues of Treatment

The assumption is that all patients and family members “want the best” for their loved one. It is reasonable to assume that the same is also true for physicians and other health care professionals. Yet when things do not work out well, one may hear a family member wonder why a certain treatment was withheld or why one treatment was chosen above another.^13^ This commonly occurs in end-of-life scenarios, when physicians may choose to find ways to withhold cardiopulmonary resuscitation (CPR), based on their understanding of EBM literature and fully understanding the limitations of what, to the public, appears to be potentially life-saving treatment. Family members may wonder why a loved one did not receive CPR in order to “bring them back from death” without adequately understanding what CPR can and cannot do, and when it might be potentially life-saving, rather than, as erroneously believed by many, “resurrecting.”^13^,^14^

This procedure is often requested by patients’ families despite clear explanations by physicians regarding the lack of benefit of CPR, its associated risks, and the related indignities as an end-of-life intervention. The main culprits for this misunderstanding, as described in the medical literature, are television medical programs that grossly exaggerate the salvation from death provided by CPR.^15^ It can be very hard for physicians to persuade devoted families to forgo this procedure when the futility of such procedures is clear—death is the expected outcome, and considerations include the resultant last indignity to a dying person versus the potential for legal action against a physician or a hospital for failure to provide CPR. A lawsuit was recently filed in Toronto that involved a 94-year-old woman who had suffered from a series of serious medical conditions. The family accused the physician and the hospital of “wrongful death, abuse of power, negligence and breach of fiduciary duties,” and is seeking $1.2 million in damages for four members of the family.^16^

## CONTRIBUTORS TO DOUBT, LOSS OF CONFIDENCE, AND PUBLIC SKEPTICISM

In light of the above, medical professionals and organized medical bodies stand to benefit from an objective analysis of the reasons for a skeptical public. The question to be asked of the medical profession, the pharmaceutical industry, and governmental and non-government health care agencies is this: Why has there been a breakdown in trust towards those providing medical care at the micro and macro levels? To answer this question, we must avoid the gut reaction of solely blaming conspiracies, the skeptical public, and groups of disgruntled patients. It is imperative to properly counter and honestly address those whose primary desire seems to be to damage the “good name” and “good reputations” of physicians, pharmaceutical industrial leaders, and practitioners.^17^,^18^

Unfortunately for all involved, medicine has a long history of dubious claims that have intentionally misled members of the public.^19^,^20^ Medical practitioners and systems have not always been transparent regarding bona-fide errors of judgement, or recommendations that posed harm to the public or individual patients, thereby sullying the reputation of the profession as a whole. The history of medicine is replete with negatively characterized “quacks” who used the guise of reputable medicine to provide ineffective and at times severely dangerous treatments to the believing and unknowing public. As noted in an internet overview of the history of medical quackery,

For thousands of years, there wasn’t much of a difference between scientific medical practices and medical quackery. The world was flat, the sky was poked full of holes and your diseases were caused by demons inside of you. There were many, many opinions on how to get those demons out … Sometimes the practitioners believed in the miracle cures being touted, and sometimes fame and acclaim were the motivating factors (the money was just a nice benefit).^21^

Of contemporary interest are the charges against Dr Mehmet Oz, a renowned cardiac surgeon from Colombia University and television personality, who has been accused of being a modern “quack” for financial and “fame” benefits. One critic of his methods wrote about his program:

*The Dr Oz Show* for the sheer magnitude of bad health advice it consistently offers, all the while giving everything a veneer of credibility. Yet, this is a symbiotic relationship. Oz needs products that excite his audience. After all, everything is a “miracle” to Oz: he’s found 16 so far.^22^

There has even been a recent call from members of the academic medical community in the United States calling on the University to sever its relationship to him.^23^

The media is replete with cases that reflect physicians who are willing in essence to “fabricate or at least exaggerate” their research findings to promote their own stature in the medical profession. This has resulted in sometimes horrendous consequences for societal beliefs and trust in important aspects of health care delivery and public policy. This has already been noted above with regard to the anti-vaccination movement—which remains strong despite public retraction of the publication by the physician involved.^24^

### The Pharmaceutical Industry, Medical Research Misrepresentation, and Public Mistrust

The false claims and fraudulent actions by some of the world’s largest and most prestigious pharmaceutical companies represent some of the greatest blemishes on the health care industry.^25^ Unfortunately, physicians may be complicit with these actions. Profits to the pharmaceutical industry outpace the huge settlements often handed out, and this compromises the public’s trust in those companies and the industry in general. The extent of such fraud is great, with many major international companies being involved in one scandal or another.^26^

Other examples of actions that undermine the public’s inherent trust in physicians and medical research efforts abound. Dr Scott Reuben, a highly respected pain researcher, was charged with fraudulently fabricating research test results.^27^ In a similar case, breast cancer research data were fraudulently falsified by Dr Roger Poisson of Montreal. Fortunately for those living with breast cancer, removing all his corrupt data from the research data pool did not negate the important findings of the study in which he was a senior participant.^28^ Despite commitments to correct procedures to reduce or eliminate research fraud, these unfortunate acts of misconduct continue to cast a pall on medical research.^29^

## WHAT DOES THE FUTURE HOLD?

One might surmise that the current state of conflict, suspicion, complaints, and litigation is untenable and could potentially negatively impact medical practice as well as the services made available to patients. If, for example, the standard of the “perfect child” becomes the measure for giving birth, the human resources available to pregnant women, be they physicians, nurses, or midwives, could well erode if they avoid or leave the profession. With obstetric malpractice rates and complaints already rising, it is not difficult to imagine fewer people choosing this field of work—despite the joy of assisting a new life into the world, one less-than-perfect baby could ruin a practitioner’s career.^30^,^31^

Such a scenario becomes likely if there are no limitations to the liability of medical professionals in the eyes of those who are dissatisfied with their health care. Simplification of regulatory measures may be helpful, as well as limiting the nature and type of liability in medical cases—for example by reducing the financial damage incurred by such lawsuits. New methods of restitution should be developed that pool resources for compensation rather than focusing on individual practitioners as the one “at fault,” and therefore the one from whom, or on whose behalf, compensation is provided. Options for fault-finding as a basis of compensation versus a no-fault system is an area of current intense interest, with risks and benefits to each model proposed, explored, or practiced worldwide.^32^

Since ethics is often closely related to science and medicine, it is worth making a robust course on ethics mandatory for all medical students. This strategy could better prepare them for the nuances and complex challenges that could be faced in the real world.^33^ Students of the health care profession are demonstrating an increased awareness of some of the profound ethical challenges that exist and that they may face as they start their careers.^34^ Although there have been improvements and enhancements to ethics education in medical schools, there is still much to be done.^35^ As Carrese et al. point out:

… despite the development of standards, milestones, and competencies related to professionalism, there is no consensus about the specific goals of medical ethics education, the essential knowledge and skills expected of learners, the best pedagogical methods and processes for implementation, and optimal strategies for assessment. Moreover, the quality, extent, and focus of medical ethics instruction vary, particularly at the graduate medical education level.^36^

The current dynamic of dissatisfaction, suspicion, and collecting conspiracy theories, which undermines the traditional trust between patients and doctors, must be addressed if the medical profession is to continue to be respected and pursued by those who indeed feel called to serve as members of the “healing profession.”

## CONCLUSION

There appears to be gradual erosion in the centuries-old respect, trust, and belief in the beneficence that motivates physicians and other health care professionals to provide the best of medical treatment to individuals and the population as a whole. The result is a disconcerting dissonance between the public and medical practitioners who are truly seeking to improve the overall quality of medical care via evidence-based medicine. Manifestations of this erosion of the traditional respect and trust are demonstrated in many ways, with dissatisfaction ultimately being expressed via complaints to regulatory bodies or by litigation. The method chosen depends more on the organizational system in place rather than the nature of the perceived inadequacy of care.

This growing mistrust ultimately undermines the patient–doctor relationship, as well as the public’s perspective of health care professionals and the system in general. This dissonance may ultimately lead to estrangement and create tension between those providing medical care and those seeking help for their medical needs. If left unresolved, the future of health care will become increasingly onerous for those wishing to enter its professions, ultimately impacting those in need of medical services.

## Abbreviations

CPR: cardiopulmonary resuscitation
EBM: evidence-based medicine
ICU: intensive care unit
MRI: magnetic resonance imaging
PSW: personal support worker
SDM: substitute decision-maker.
